# The impact of community-acquired pneumonia on the health-related quality-of-life in elderly

**DOI:** 10.1186/s12879-017-2302-3

**Published:** 2017-03-14

**Authors:** Marie-Josée J. Mangen, Susanne M. Huijts, Marc J. M. Bonten, G. Ardine de Wit

**Affiliations:** 1 Julius Center for Health Sciences and Primary Care, University Medical Center Utrecht, Heidelberglaan 100, 3584 CX Utrecht, The Netherlands; 2 Department Respiratory Medicine, University Medical Center Utrecht, Utrecht, The Netherlands; 3 Department of Medical Microbiology, University Medical Center Utrecht, Utrecht, The Netherlands

**Keywords:** Quality-of-life, Community-acquired pneumonia, Elderly, Follow-up, Mortality

## Abstract

**Background:**

The sustained health-related quality-of-life of patients surviving community-acquired pneumonia has not been accurately quantified. The aim of the current study was to quantify differences in health-related quality-of-life of community-dwelling elderly with and without community-acquired pneumonia during a 12-month follow-up period.

**Methods:**

In a matched cohort study design, nested in a prospective randomized double-blind placebo-controlled trial on the efficacy of the 13-valent pneumococcal vaccine in community-dwelling persons of ≥65 years, health-related quality-of-life was assessed in 562 subjects hospitalized with suspected community-acquired pneumonia (i.e. diseased cohort) and 1145 unaffected persons (i.e. non-diseased cohort) matched to pneumonia cases on age, sex, and health status (EQ-5D-3L-index). Health-related quality-of-life was determined 1–2 weeks after hospital discharge/inclusion and 1, 6 and 12 months thereafter, using Euroqol EQ-5D-3L and Short Form-36 Health survey questionnaires. One-year quality-adjusted life years (QALY) were estimated for both diseased and non-diseased cohorts. Separate analyses were performed for pneumonia cases with and without radiologically confirmed community-acquired pneumonia.

**Results:**

The one-year excess QALY loss attributed to community-acquired pneumonia was 0.13. Mortality in the post-discharge follow-up year was 8.4% in community-acquired pneumonia patients and 1.2% in non-diseased persons (*p* < 0.001). During follow-up health-related quality-of-life was persistently lower in community-acquired pneumonia patients, compared to non-diseased persons, but differences in health-related quality-of-life between radiologically confirmed and non-confirmed community-acquired pneumonia cases were not statistically significant.

**Conclusions:**

Community-acquired pneumonia was associated with a six-fold increased mortality and 16% lower quality-of-life in the post-discharge year among patients surviving hospitalization for community-acquired pneumonia, compared to non-diseased persons.

**Trial registration:**

ClinicalTrials.gov, NCT00812084.

**Electronic supplementary material:**

The online version of this article (doi:10.1186/s12879-017-2302-3) contains supplementary material, which is available to authorized users.

## Background

Community-acquired pneumonia (CAP) causes considerable disease and economic burden. In the Netherlands, the overall incidence of CAP was estimated to be 295 per 100,000 inhabitants, yielding approximately 50,000 episodes per year, with considerable variation between age-groups [1]. Approximately 45% of all CAP episodes occur in persons aged ≥65 years [1]. In the period after recovery, CAP is associated with higher risks on e.g. stroke and other cardiovascular events [2, 3]. Both CAP and the occurrence of these other diseases in the post-discharge period may impact on the health-related quality-of-life (HrQol). However, only limited data are available on HrQol after a CAP episode [4]. In only two of six studies that focused on HrQol after CAP [5–10] patient follow-up exceeded 6 weeks. El Moussaoui et al. [7] followed patients for 18 months using the Short-Form (36) Health survey [11] (referred hereafter as SF36), and Honselmann et al. [10] determined the HrQol at one-year post-discharge using the Euroqol EQ-5D-3L instrument [12] (referred hereafter as EQ5D) in patients that had survived an episode of pneumonia and/or sepsis for which admission to intensive care was needed. These studies were all descriptive, and none of these studies quantified the excess quality-adjusted life-years (QALY) lost due to CAP in comparison with non-diseased persons (i.e. no pneumonia). The aim of the current study was to quantify differences in HrQol of community-dwelling elderly with and without CAP during a 12-month follow-up period. In addition, possible HrQol differences between radiologically confirmed and radiologically non-confirmed CAP cases were investigated.

## Methods

### Study design, setting and participants

**Fig. 1:**
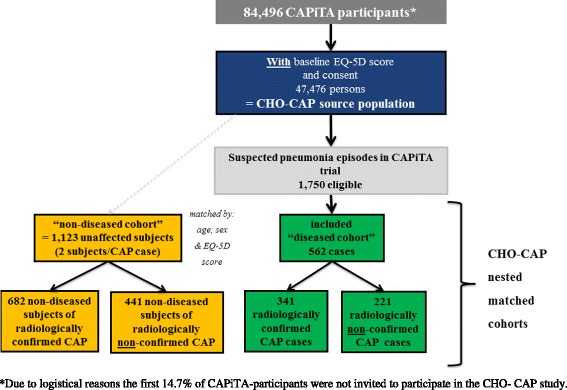
Flow chart of CHO-CAP study. *Due to logistical reasons the first 14.7% of CAPiTA-participants were not invited to participate in the CHO-CAP study

The current study, “Costs, Health status and Outcomes of CAP (Community-Acquired Pneumonia)” (CHO-CAP), was executed in parallel to the “Community-Acquired Pneumonia Immunization trial in Adults” (CAPiTA) trial, a placebo-controlled double-blinded RCT evaluating the effectiveness of a 13-valent pneumococcal conjugate vaccine in 84,496 community-dwelling elderly in the Netherlands [13, 14]. The CHO-CAP-study used a nested matched-cohort study design when recruiting patients hospitalized with a clinical suspicion of a pneumonia episode from the CAPITA-study population and prospectively followed them, along with non-diseased subjects (i.e. no pneumonia), for a one-year post-discharge period (Fig. 1) [15]. CAPiTA-participants were approached for study participation in CHO-CAP at the time of vaccination (November-2008-January-2010). Overall, 72,074 CAPiTA-participants^1^ received the invitation to return the CHO-CAP-baseline questionnaire together with a signed informed consent form. Those who returned both questionnaire and informed consent formed the CHO-CAP source population (*n* = 47,476^2^). This source population was a priori eligible for the nested matched-cohort study. Within the CAPiTA-trial, 3225 patients with a suspected pneumonia were identified in 56 Dutch sentinel hospitals. Potential cases for the “diseased”-cohort were subjects with a first-time suspected pneumonia episode, participating in the CHO-CAP source population, without recently diagnosed malignancy and able to complete questionnaires. After hospital discharge, these subjects were invited for participation in the diseased cohort. For feasibility reasons, we included all patients hospitalized with a clinical suspicion, which were later stratified upon either or not receiving a radiological confirmation of CAP by the blinded chest X-ray adjudication committee. Those who consented were visited at home within 2 weeks of hospital discharge by trained interviewers. They were asked to complete self-administered questionnaires during the home visit (day 0), and at 1, 6 and 12 months after the home visit through postal questionnaires. For each subject in the diseased cohort, two matched non-diseased subjects (i.e. no pneumonia) were identified from the CHO-CAP source population (referred hereafter as non-diseased subjects). Matching was based on age, sex, and EQ-5D-3L-index collected at vaccination, implying that health status of people with suspected pneumonia and their matched non-diseased subjects was similar at the time of vaccination. Non-diseased subjects adhered to the same inclusion criteria as suspected pneumonia patients. They were asked to complete self-administered questionnaires during the home visit (day 0). Further questionnaires were sent by regular mail 1, 6 and 12 months after the home visit. For full details see Mangen et al. [15].

### Definitions of subgroups

Additionally, we did distinguish between “*radiologically confirmed*” CAP cases and the matched non-diseased subjects and “*radiologically* non-*confirmed*” CAP cases and their matched non-diseased subjects. A “*radiologically confirmed*” CAP was defined as the presence of two or more clinical signs of pneumonia together with a chest x-ray consistent with pneumonia, identical to the definition used within the CAPiTA-trial [13].

### Data collection

Date of birth, sex, place of residence, loss-to-follow-up due to death during the follow-up period and causes of death were extracted from the CAPiTA-study files [14]. Health status (EQ5D) and socio-demographic status (living situation and education) were collected at the time of vaccination with the CHO-CAP-baseline questionnaire. Full details of data collection of nested matched-cohort study are provided in Mangen et al. [15]. In short, comorbidity details were collected during the home visit. Current living situation was collected at all four contact moments. Health status (EQ5D) was collected thrice for suspected pneumonia cases during the home-visit interview, reflecting health status (1) at day of interview, (2) at the worst moment during the recent pneumonia episode, and (3) previous to the recent pneumonia episode. Health status was also collected at month 1, month 6 and month 12 after initial visit using both EQ5D and SF-36. For non-diseased subjects, both EQ5D and SF-36 were administered at all four contact moments. For suspected pneumonia cases, clinical information on hospital admission (e.g. X-ray result; clinical symptoms; length of stay) was extracted from the CAPiTA-study files [14].

### Health status questionnaires

The SF-36 is composed of 36-items measuring health across eight domains (physical functioning, social functioning, role limitations with respect to physical activities, role limitations with respect to emotional activities, pain, mental health, vitality and general health perception). Responses to each item within a domain are combined to generate a score from 0 to 100, where 100 indicates best health [11]. Because of the elderly population - some potentially in poor health - the usual order of items in question 3 was inversed [16]. Applying the scoring-method developed by Brazier et al. [17], we further derived the SF-6D health-index from the SF-36 survey, a numerical index between 0 (“death”) and 1 (“full health”) [17]. The EQ5D consists of two parts, the EQ-5D-descriptive system and the EQ-visual analogue scale (VAS) [18]. The EQ-VAS records the participants’ self-reported health on a VAS from 0 (“Worst imaginable health state”) to 100 (“Best imaginable health state”) [18]. The EQ5D-descriptive system consists of five domains (mobility, self-care, usual activities, pain/discomfort and anxiety/depression) and three functioning levels (no problems, some problems or severe problems) [12, 18]. The EQ5D health states were scored with the Dutch value-set [19], to obtain EQ-5D-3L summary indexes (EQ-index) ranging from 0 (“death”) to 1 (“full health”) [12, 19].

### QALY estimations

Quality Adjusted Life Years (QALY) is a concept used to reflect a year in full quality of life. Hence, QALYs combine both length of life and quality of life. Quality of life is represented in a value between 0 (death) and 1 (optimal quality of life). QALYs are estimated by multiplying length of life with the indicator value for quality of life. One-year QALY estimates, with and without pneumonia episode included, were calculated for both cohorts, using the self-reported EQ5D health states and its associated index values at the different contact moments based on recorded date of contact moment. An area under the curve approach was followed by interpolating between the observations provided by the patients. For patients who died, we calculated QALY by using the date of death and a utility score of 0 from that date onwards. For missing EQ-indexes, ten imputations were performed. QALYs were calculated in each imputed dataset and averaged over the ten imputated datasets. The observed utility difference between both cohorts was attributed to the CAP episode. Excess QALY loss was calculated for the one-year post-discharge period, excluding and including the CAP episode, respectively (see Additional file 1: Figure S1A for illustration). Additionally, we estimated the QALY and the excess QALY loss for one-year survivors. Pairs of pneumonia cases and non-diseased subjects in which one of the three died during the follow-up were excluded from these estimates.

### Data analysis

Health status and QALY estimates in the suspected pneumonia cases and non-diseased subjects are presented for the different follow-up moments, in a decomposed manner (i.e. at the level of the different domains of quality of life) and as summary index. Causes of death were categorized into five categories: (1) infectious diseases; (2) chronic lung diseases; (3) cancer; (4) cardiovascular events and stroke and (5) others. Depending on the nature and distribution of data, we used Chi-square test for categorical variables (e.g. sex, education) and non-parametric tests for non-normally distributed variables (e.g. scores) to test for differences between both cohorts (i.e. diseased and non-diseased cohort) and for differences between patients with and without confirmed CAP. Correlation between the different instruments was studied using Spearman’s rank correlation coefficient. All analyses were performed using IBM SPSS Statistics version 21.

## Results

### Study participants

Of the 3225 identified suspected pneumonia episodes in the CAPiTA-trial, 1750 (54%) belonged to the CHO-CAP source population, of which 562 (32%) participated; 341 (61%) had radiologically confirmed CAP and 221 (39%) had radiologically non-confirmed CAP (Fig. 1). Reasons for non-participation/exclusion are provided in in the Additional file 1: Table S1A.

### Suspected pneumonia cases

Baseline characteristics of the suspected pneumonia cases and their non-diseased subjects are summarized in Table 1. Despite adequate matching on three criteria, non-diseased subjects had fewer comorbidities, were higher educated and were more often from the south of the Netherlands than pneumonia cases (Table 1).

**Table 1:** Baseline characteristics of suspected pneumonia cases and their non-diseased subjects

	Suspected pneumonia cases (i.e. “diseased” cohort^b^)	Non-diseased subjects	*p*-value
Episodes/subjects	562	1123	
Matching criteria			
Male, in %	71.0	71.1	ns
Age at inclusion^a^, median (IQR)	76 (72–82)	76 (72–81)	ns
EQ5D-index (at vaccination), median (IQR)	0.89 (0.78–1.00)	0.89 (0.78–1.00)	ns
Other characteristics			
Number of self-reported comorbidities at inclusion^a^, median (IQR)	2 (1–4)	2 (1–3)	<0.001
Educational level, in %			<0.001
Low	53.6	36.3	
Medium	28.3	36.4	
High	17.4	27.0	
Missing	0.7	0.3	
Region, in %			<0.001
North	3.4	4.0	
East	27.4	18.4	
West	32.2	24.1	
South	37.0	53.4	
Living situation at vaccination, in %			ns
Single household	27.6	26.8	
Two or more person/household	71.5	72.8	
Elderly home	0.7	0.4	
Missing	0.2	0.1	
Vaccinated, in %	48.4	51.6	ns

*Abbreviations*: *SD*, Standard deviation, *ns* not significant

^a^At inclusion in cohort (= day of home visit). ^b^Composed of radiologically confirmed CAP cases (i.e. having a positive X-rays and at least 2 clinical criteria) and radiologically non-confirmed CAP cases

Compared to the non-diseased subjects, suspected pneumonia cases more frequently died during the one-year follow-up period (8.4% vs 1.2%;*p* < 0.001 (in the Additional file 1: Figure S2A.); attributable risk:0.059(95% CI:0.058–0.060)) or withdrew from the study (16.7% vs 9.9%; *p* < 0.001), mainly because of bad health (in the Additional file 1: Table S2A). Chronic lung diseases, cardiovascular events and stroke were more frequently reported as being the cause of death for suspected pneumonia cases than for non-diseased subjects (*p* = 0.054; in the Additional file 1: Table S2A)).

**Fig. 2:**
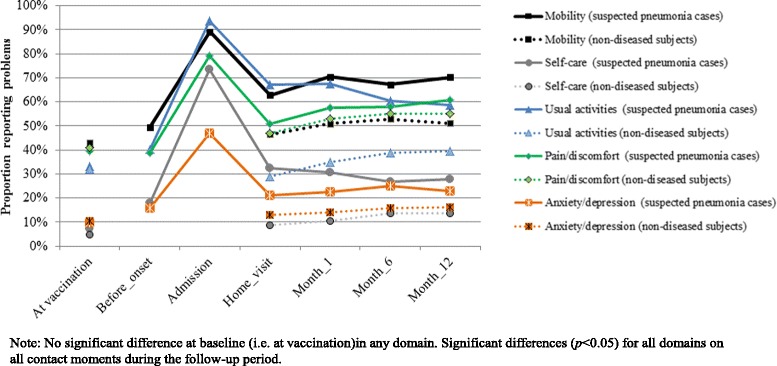
Profile of the population, using the EQ5D instrument: Percentage reporting any problems per domain at different contact moments for the suspected pneumonia cases and the non-diseased subjets, respectively. Note: No significant difference at baseline (i.e. at vaccination) in any domain. Significant differences (*p* < 0.05) for all domains on all contact moments during the follow-up period

**Fig. 3:**
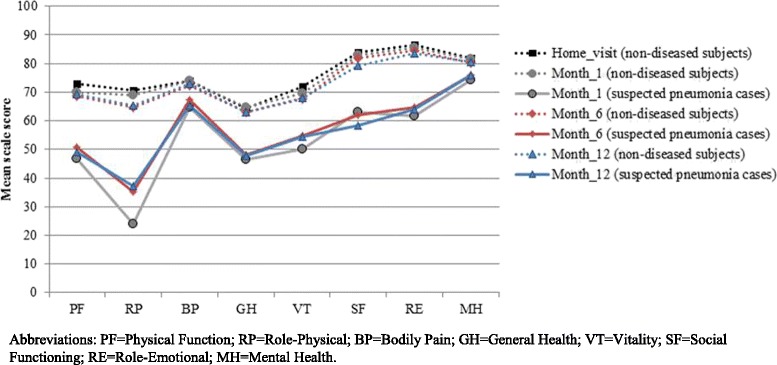
SF-36 mean scale scores at different contact moments for the suspected pneumonia cases and the non-diseased subjets, respectively. Abbreviations: PF = Physical Function; RP = Role-Physical; BP = Bodily Pain; GH = General Health; VT = Vitality; SF = Social Functioning; RE = Role-Emotional; MH = Mental Health

Figure 2 shows the distribution of suspected pneumonia cases and non-diseased subjects reporting problems in the five domains of the EQ5D-instrument at all observation moments. By definition, non-diseased subjects and suspected pneumonia cases had a similar health status at the moment of vaccination. However, during follow-up suspected pneumonia cases more frequently reported problems in the five domains of the EQ5D-instrument than non-diseased subjects at all contact moments and for all domains (Fig. 2). This finding was confirmed by the SF36-questionnaire. Suspected pneumonia cases had persistently lower SF-36 mean scale scores on all domains and during all contact moments, compared to their non-diseased subjects (Fig. 3). Furthermore, HrQol, as expressed in EQ5D and SF-6D index value and EQ-VAS score of suspected pneumonia cases was constantly lower than HrQol of non-diseased subjects during the one-year follow-up (Table 2, in the Additional file 1: Figure S3A). At month-12, HrQol-scores were significantly lower for survivors in the suspected pneumonia cohort compared to the non-diseased cohort (EQ5D-index:0.74 vs 0.82(*p* < 0.001) and SF6D-index:0.68 vs 0.75(*p* < 0.001)).

**Table 2:** EQ5D-index, EQ-VAS and SF6D-index for suspected pneumonia and the non-diseased subjects

	Suspected pneumonia cases		Non-diseased subjects		*p*-value
	Mean (SD)/Median (IQR)	Missing/died	Mean (SD)/Median (IQR)	Missing/died	
EQ5D-index					
At vaccination^a^	0.87 (0.16)/0.89 (0.78–1.00)	0/0	0.87 (0.16)/0.89 (0.78–1.00)	0/0	ns
Prior to illness onset	0.81 (0.23)/0.86 (0.78–1.00)	0/0	-	-	-
At admission	0.23 (0.32)/0.24 (−0.00–0.43)	0/0	-	-	-
During home visit	0.70 (0.26)/0.78 (0.52–0.89)	0/0	0.84 (0.18)/0.84 (0.78–1.00)	0/0	<0.001
Month 1	0.72 (0.24)/0.78 (0.65–0.89)	60/4	0.83 (0.17)/0.84 (0.78–1.00)	44/2	<0.001
Month 6	0.74 (0.23)/0.78 (0.66–0.89)	85/29	0.82 (0.18)/0.84 (0.78–1.00)	76/4	<0.001
Month 12	0.74 (0.23)/0.78 (0.67–0.89)	104/49	0.82 (0.18)/0.84 (0.78–1.00)	124/14	<0.001
EQ5D-VAS					
Prior to illness onset	71 (15.3)/70 (60–80)	0/0	-	-	-
At admission	33 (16.0)/30 (20–41)	1/0	-	-	-
During home visit	62 (16.7)/65 (50–70)	0/0	76 (13.2)/80 (70–85)	3/0	<0.001
Month 1	64 (16.3)/65 (50–75)	62/4	76 (13.9)/79 (70–85)	37/2	<0.001
Month 6	65 (16.4)/70 (55–78)	86/29	74 (15.2)/75 (65–84)	73/4	<0.001
Month 12	64 (17.5)/69 (50–78)	98/49	75 (14.6)/75 (68–85)	122/14	<0.001
SF6D-index					
During home visit	-	-	0.77 (0.13)/0.79 (0.66–0.88)	9/0	-
Month 1	0.65 (0.13)/0.63 (0.58–0.73)	81/4	0.76 (0.13)/0.77 (0.65–0.88)	69/2	<0.001
Month 6	0.68 (0.14)/0.66 (0.59–0.79)	99/29	0.75 (0.14)/0.75 (0.63–0.88)	116/4	<0.001
Month 12	0.68 (0.14)/0.66 (0.58–0.77)	122/49	0.75 (0.13)/0.75 (0.63–0.87)	149/14	<0.001

*Abbreviations*: *SD* Standard deviation, *ns* not significant

^a^matching criterion

One-year QALY estimates, excluding the CAP episode and using the self-reported EQ5D health status and its associated-index values, were 0.68 and 0.81 for suspected pneumonia cases and non-diseased subjects (*p* < 0.001), yielding a utility difference between both cohorts of −0.13 attributable to suspected pneumonia. One-year QALY estimates, including the CAP episode, were 0.67 and 0.81 for suspected pneumonia cases and non-diseased subjects (*p* < 0.001), yielding an excess QALY loss of 0.15. Slightly smaller QALY differences were obtained when considering only survivors (Table 3), resulting in excess QALY loss of 0.10, if excluding the CAP episode, and 0.11, if including the CAP episode, respectively.

**Table 3:** Utility difference attributable to suspected pneumonia

	QALY suspected pneumonia case (SE)	QALY non-diseased subjects (SE)	Utility difference attributable to suspected pneumonia
All cases			
One-year post-discharge	0.68 (0.01)	0.81 (0.01)	−0.13
Pneumonia episode & one-year post-discharge	0.67 (0.01)	0.81 (0.01)	−0.15
Only survivors			
One-year post-discharge	0.72 (0.01)	0.82 (0.01)	−0.10
Pneumonia episode & one-year post-discharge	0.71 (0.01)	0.82 (0.01)	−0.11

*Abbreviations*: *QALY* quality-adjusted life years, *SE* standard error

EQ5D-index, EQ-VAS and SF6D-index were positively correlated at all contact moments for suspected pneumonia cases and non-diseased subjects (rho > 0.45), in the Additional file 1: Table S3A and Table S4A. The highest correlation was found between EQ5D-index and SF6D-index for both suspected pneumonia cases and non-diseased subjects (rho >0.67 for all contact moments).

### Radiologically confirmed and non-confirmed CAP cases

There were apparent differences between patients with radiologically confirmed and non-confirmed CAP, but when repeating analyses in a stratified manner interpretation did not change. Radiologically confirmed and non-confirmed CAP cases did not differ in baseline characteristics and were adequately matched to their non-diseased subjects (in the Additional file 1: Table S5A). Radiologically confirmed CAP cases stayed significantly longer in hospital than radiologically non-confirmed CAP cases, but had comparable median numbers of clinical symptoms, pneumonia severity index (PSI) scores [20], admissions to ICU and readmissions within 30 days, Table 4. Mortality was higher for patients with radiologically confirmed CAP (10.3%) compared to non-confirmed CAP (5.4%; *p* = 0.043), but the causes of death were comparable (in the Additional file 1: Table S6A). The attributable risks of dying, compared to non-diseased subjects, were 0.083 (95%CI:0.082–0.084) for those with radiologically confirmed CAP and 0.047 (95%CI:0.047–0.048) for those with non-confirmed CAP. As compared to their non-diseased subjects, both radiologically confirmed and non-confirmed CAP cases had a significantly persistent lower HrQol, independent of the instrument used (see in the Additional file 1: Figure S4A-S6A and Table S7A.). The excess QALY loss attributable to CAP was with 0.14 for radiologically confirmed CAP cases and 0.12 for radiologically non-confirmed CAP cases, of the same magnitude, independent of radiological confirmation of CAP (in the Additional file 1: Table S8A.).

**Table 4:** Clinical data of radiologically confirmed and radiologically non-confirmed CAP cases

	Radiologically confirmed CAP cases	Radiologically non-confirmed CAP cases	*p*-value
Episodes/subjects	341	221	
Positive chest X-ray, in %	100	0	^a^
Number of clinical criteria, median (IQR)	4 (3–5)	4 (3–5)	ns
PSI score, median (IQR)	93 (80–111)	90 (78–108)	ns
Admitted to ICU, in %	9.7	5.2	ns
Readmitted within 30 days, in %	5.0	3.6	ns
LOS in days, median (IQR)			
At 1st admission,	8 (6–12)	7 (5–11)	0.006
Including readmission	8 (6–11)	7 (5–10)	0.011

*Abbreviations*: *IQR* interquartile range, *ns* not significant

^a^According to definition radiologically confirmed CAP cases had to have a positive X-rays

## Discussion

In immunocompetent elderly, hospitalization for suspected pneumonia was associated with a six-fold higher risk of mortality and an average loss of QALYs attributable to pneumonia of 0.13 after 1 year, compared to non-diseased subjects. Patients with radiologically confirmed CAP had a two-fold higher mortality risk than those with radiologically non-confirmed CAP, but the average loss of QALYs attributable to CAP among survivors was comparable. The one-year QALY loss associated with a CAP episode (0.13, excluding the CAP episode, and 0.15, including the CAP episode) is two-fold higher than the QALY loss that we used [21] in a cost-effectiveness analysis of pneumococcal vaccination (0.071) and is more than tenfold higher than the QALY loss used by Melegaro and Emunds [22] in 2004 and in most cost-effectiveness studies conducted thereafter in Western European countries (e.g. [23–26]), namely 0.004 for inpatient CAP and 0.0079 for bacteraemia. These estimates of QALY loss were based on expert opinion and not real-life data. Preventing a higher QALY loss through pneumococcal vaccination by definition contributes to more favourable cost-effectiveness of vaccination. For example using a QALY loss of 0.15 rather than 0.07 would have resulted in a somewhat more favourable incremental cost-effectiveness ratio of 8,200 €/QALY versus the 8,650 €/QALY presented in our recently published cost-effectiveness analysis [21]. Although the difference in QALYs is relatively large, the impact on the cost-effectiveness ratio is relatively limited, as mortality has a much higher impact on the cost per QALY gained than quality-of-life.

Strengths of this study include the rigid prospective study design nested within a randomized double-blinded placebo-controlled trial that created the possibility to quantify the excess QALY lost due to CAP in community-dwelling elderly using a one-year follow-up period. We also conducted separate analyses for patients with radiographically confirmed CAP and those without confirmation.

To control for potential biases between pneumonia cases and non-diseased subjects, subjects in both cohorts were matched on age, sex and EQ5D-index as collected at the time of vaccination. CAP patients had slightly more comorbidities and a lower educational level, factors known to be negatively associated with health status [27–29]. The calculated excess QALY loss might therefore be a slight overestimation of the attributable QALY loss. Furthermore, our study may have suffered from a healthy participant effect, as the study population consisted of subjects that were willing-to-participate in a one-year follow-up study who may have been healthier than the non-responding CAP patients. Indeed, one of the major arguments to refuse participation was self-perceived bad health, and patients with a recent cancer diagnosis were excluded as well. In a sicker population, mortality attributable to CAP would most likely have been higher. As a result, the observed QALY loss attributable to CAP and the mortality risk within the first year post-discharge may have been underestimated.

## Conclusion

The current study is the first that provides detailed HrQol during the recovery process of hospitalized elderly suspected pneumonia patients for a one-year post-discharge period using the EQ5D and SF36 questionnaires. It further provides a QALY loss attributable to CAP for community-dwelling elderly, which is a necessity for economic analyses targeted at preventing pneumonia infections and as such contributes to more realistic future estimates of cost-effectiveness of preventive interventions for this infection. The CAP episode is the onset of sustained loss of quality-of-life, with an estimated difference in QALY of 16–18% between CAP patients and their non-diseased subjects.

## Additional file

Additional file 1:
**Figure S1A.** Observed EQ-5D indexes for suspected pneumonia cases and non-diseased subjects during the one-year post-discharge period, excluding the CAP episode. **Figure S2A.** Survivors (%) in the diseased cohort (i.e. suspected pneumonia cases) and the non-diseased cohort during the one-year follow-up. **Figure S3A.** Mean EQ-5D-3 L-index, EQ-VAS and SF6D-index at different contact moments for the suspected CAP cases and the non-diseased subjects, respectively. **Figure S4A.** Profile of the population using EQ5D-instrument: Percentage reporting any problems per domain at different contact moments for A) the radiologically confirmed CAP cases and their non-diseased subjects, and B) the radiologically non-confirmed CAP cases and their non-diseased subjects, respectively. **Figure S5A.** SF-36 mean scale scores at different contact moments for A) the radiologically confirmed CAP cases and their non-diseased subjects, and B) the radiologically non-confirmed CAP cases and their non-diseased subjects, respectively. **Figure S6A.** Mean EQ-5D-3 L-index, EQ-VAS and SF6D-index at different contact moments for the radiologically confirmed CAP cases and their non-diseased subjects (A), and for the radiologically non-confirmed CAP cases and their non-diseased subjects (B). **Table S1A.** Exclusion criteria and reasons for nonparticipation in the “diseased” cohort of eligible suspected pneumonia episodes. **Table S2A.** Living situation, loss-to-follow up and deaths of suspected pneumonia cases and non-diseased subjects during the one-year follow-up. **Table S3A.** Spearman’s rho for EQ-VAS, EQ5D-index and SF6D-index at the different contact moments for suspected pneumonia cases. **Table S4A.** Spearman’s rho for EQ-VAS, EQ5D-index and SF6D-index at the different contact moments for non-diseased subjects. **Table S5A.** Baseline characteristics of radiologically confirmed and non-confirmed CAP cases and their non-diseased subjects. **Table S6A.** Living situation, loss-to-follow up and mortality of radiologically confirmed and non-confirmed CAP cases and their non-diseased subjects during the one-year follow-up. **Table S7A.** EQ5D-index, EQ-VAS and SF6D-index for the radiologically confirmed and non-confirmed CAP cases and their non-diseased subjects. **Table S8A.** Utility differences attributable to radiologically confirmed CAP and radiologically non-confirmed CAP, respectively. (DOCX 602 kb)

## Abbreviations

CAP: Community-acquired pneumonia
CAPiTA: Community-acquired pneumonia immunization trial in adults
CHO-CAP: Costs, health status and outcomes of CAP (Community-Acquired Pneumonia) - study
EQ5D: Euroqol EQ-5D-3L instrument
HrQol: Health-related quality-of-life
QALY: Quality-adjusted life years
SD: Standard deviation
SF36: Short form-36 health survey questionnaire
VAS: Euroqol visual analogue scale

## Footnotes

1 Due to logistical reasons the first 14.7% of CAPiTA-participants were not invited to participate in the CHO-CAP-study.

2 48,634 participants returned the questionnaire with signed informed consent. For 47,476 participants (97%) an EQ5D-score could be estimated.
