# Supplementary material for: The impact of community-acquired pneumonia on the health-related quality-of-life in elderly
Source: BMC Infect Dis. 2017 Mar 14;17:208. doi: 10.1186/s12879-017-2302-3 (PMC5351062; doi:10.1186/s12879-017-2302-3)
Supplement: Additional file 1: — Figure S1A. Observed EQ-5D indexes for suspected pneumonia cases and non-diseased subjects during the one-year post-discharge period, excluding the CAP episode. Figure S2A. Survivors (%) in the diseased cohort (i.e. suspected pneumonia cases) and the non-diseased cohort during the one-year follow-up. Figure S3A. Mean EQ-5D-3 L-index, EQ-VAS and SF6D-index at different contact moments for the suspected CAP cases and the non-diseased subjects, respectively. Figure S4A. Profile of the population using EQ5D-instrument: Percentage reporting any problems per domain at different contact moments for A) the radiologically confirmed CAP cases and their non-diseased subjects, and B) the radiologically non-confirmed CAP cases and their non-diseased subjects, respectively. Figure S5A. SF-36 mean scale scores at different contact moments for A) the radiologically confirmed CAP cases and their non-diseased subjects, and B) the radiologically non-confirmed CAP cases and their non-diseased subjects, respectively. Figure S6A. Mean EQ-5D-3 L-index, EQ-VAS and SF6D-index at different contact moments for the radiologically confirmed CAP cases and their non-diseased subjects (A), and for the radiologically non-confirmed CAP cases and their non-diseased subjects (B). Table S1A. Exclusion criteria and reasons for nonparticipation in the “diseased” cohort of eligible suspected pneumonia episodes. Table S2A. Living situation, loss-to-follow up and deaths of suspected pneumonia cases and non-diseased subjects during the one-year follow-up. Table S3A. Spearman’s rho for EQ-VAS, EQ5D-index and SF6D-index at the different contact moments for suspected pneumonia cases. Table S4A. Spearman’s rho for EQ-VAS, EQ5D-index and SF6D-index at the different contact moments for non-diseased subjects. Table S5A. Baseline characteristics of radiologically confirmed and non-confirmed CAP cases and their non-diseased subjects. Table S6A. Living situation, loss-to-follow up and mortality of radiologically confi [file 12879_2017_2302_MOESM1_ESM.docx]

**ADDITIONAL FILE**

**Manuscript**

The impact of community-acquired pneumonia on the health-related quality-of-life in elderly

**Additional File Online Content**

**Content of Figures**

[Figure A.1 – Observed EQ-5D indexes for suspected pneumonia cases and non-diseased subjects during the one-year post-discharge period, excluding the CAP episode. 4](#_Toc472341895)

[Figure A.2 – Survivors (%) in the diseased cohort (i.e. suspected pneumonia cases) and the non-diseased cohort during the one-year follow-up. 5](#_Toc472341896)

[Figure A.3 – Mean EQ-5D-3L-index, EQ-VAS and SF6D-index at different contact moments for the suspected CAP cases and the non-diseased subjects, respectively. 7](#_Toc472341897)

[Figure A.4 – Profile of the population using EQ5D-instrument: Percentage reporting any problems per domain at different contact moments for A) the radiologically confirmed CAP cases and their non-diseased subjects, and B) the radiologically non-confirmed CAP cases and their non-diseased subjects, respectively. 14](#_Toc472341898)

[Figure A.5 – SF-36 mean scale scores at different contact moments for A) the radiologically confirmed CAP cases and their non-diseased subjects, and B) the radiologically non-confirmed CAP cases and their non-diseased subjects, respectively. 15](#_Toc472341899)

[Figure A.6 – Mean EQ-5D-3L-index, EQ-VAS and SF6D-index at different contact moments for the radiologically confirmed CAP cases and their non-diseased subjects (A), and for the radiologically non-confirmed CAP cases and their non-diseased subjects (B) 16](#_Toc472341900)

**Content of Tables**

[Table A.1 – Exclusion criteria and reasons for nonparticipation in the “diseased” cohort of eligible suspected pneumonia episodes. 3](#_Toc472341903)

[Table A.2 – Living situation, loss-to-follow up and deaths of suspected pneumonia cases and non-diseased subjects during the one-year follow-up. 6](#_Toc472341904)

[Table A.3 – Spearman’s rho for EQ-VAS, EQ5D-index and SF6D-index at the different contact moments for suspected pneumonia cases. 8](#_Toc472341905)

[Table A.4 – Spearman’s rho for EQ-VAS, EQ5D-index and SF6D-index at the different contact moments for non-diseased subjects. 9](#_Toc472341906)

[Table A.5 – Baseline characteristics of radiologically confirmed and non-confirmed CAP cases and their non-diseased subjects. 10](#_Toc472341907)

[Table A.6 – Living situation, loss-to-follow up and mortality of radiologically confirmed and non-confirmed CAP cases and their non-diseased subjects during the one-year follow-up. 11](#_Toc472341908)

[Table A.7 – EQ5D-index, EQ-VAS and SF6D-index for the radiologically confirmed and non-confirmed CAP cases and their non-diseased subjects 12](#_Toc472341909)

[Table A.8 –Utility differences attributable to radiologically confirmed CAP and radiologically non-confirmed CAP, respectively. 17](#_Toc472341910)

Table A.1 – Exclusion criteria and reasons for nonparticipation in the “diseased” cohort of eligible suspected pneumonia episodes.

| **Reason** | **Episodes, n (%)** |
| --- | --- |
| *Exclusion criterion* |  |
| CAP suspicion, but not hospitalized | 11 (0.9%) |
| Admission for a second or further pneumonia episode | 434 (36.5%) |
| Died during hospital admission | 149 (12.5%) |
| Recent cancer diagnosis | 85 (7.2%) |
| Not able to complete questionnaires | 26 (2.2%) |
| *Other reasons for exclusion* |  |
| Other formal reasons (e.g. not being able to get in contact,  discharge was confirmed too late, no contract with hospital at the time of admission) | 181 (15.2%) |
| No confirmation of CAP according to patient/local trial nurse^a^ | 85 (7.2%) |
| *Not willing to participate* |  |
| Not willing to participate | 217 (18.3%) |
| **Total** | **1,188 (100%)** |

1. Patient perception and local trial nurses judgement were not always in line with the final adjudication in the CAPiTA-study.

Figure A.1 – Observed EQ-5D indexes for suspected pneumonia cases and non-diseased subjects during the one-year post-discharge period, excluding the CAP episode.

Quality Adjusted Life Years (QALY) is a concept used to reflect a year in full quality of life. Hence, QALYs combine both length of life and quality of life. Quality of life is represented in a value between 0 (death) and 1 (optimal quality of life). QALYs are estimated by multiplying length of life with the indicator value for quality of life. One-year QALY estimate, without pneumonia episode included, was calculated for both cohorts, using the self-reported EQ5D health states and its associated index values at the different contact moments based on recorded date of contact moment (see figure). An area under the curve approach was followed by interpolating between the observations provided by the patients. The observed utility difference between both cohorts was attributed to the CAP episode.


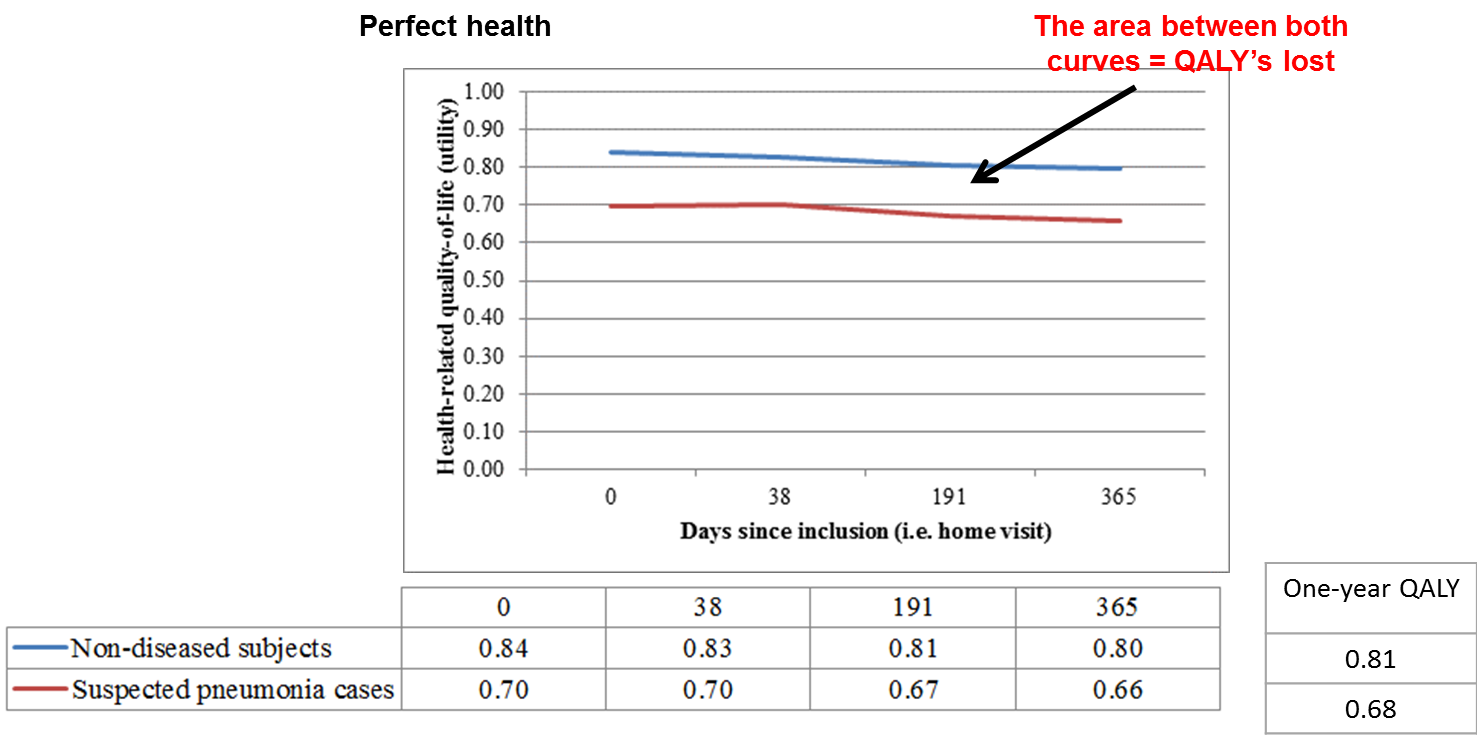


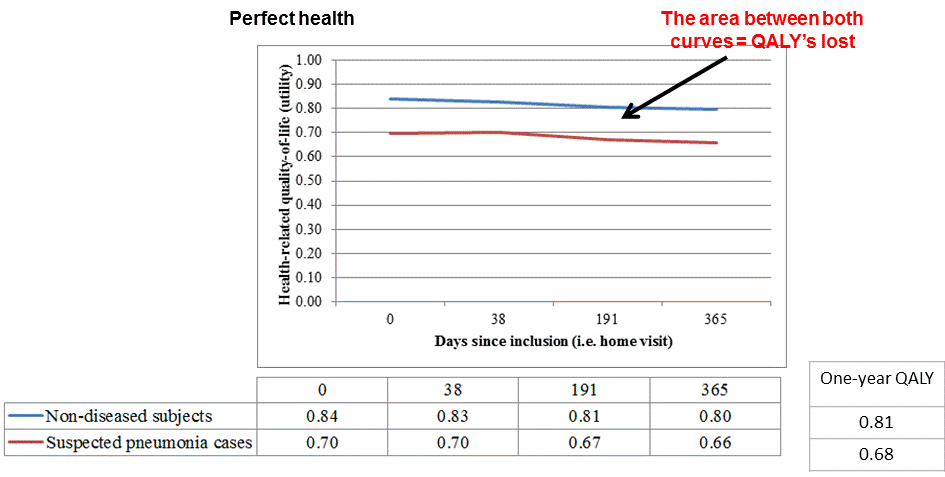


Note: The here reported EQ-5D indexes are different than the one presented in Table 2. In Table 2 we presented the “raw” reported EQ-5D indexes, whereas here imputed and averages EQ-5D indexes, which were used to estimate the excess QALY loss attributable to suspected pneumonia.

Figure A.2 – Survivors (%) in the diseased cohort (i.e. suspected pneumonia cases) and the non-diseased cohort during the one-year follow-up.


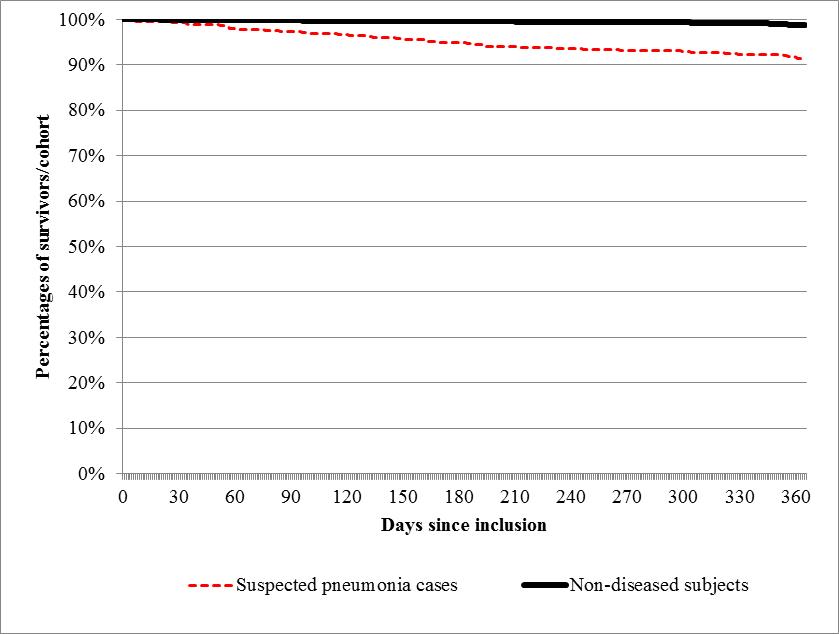


Table A.2 – Living situation, loss-to-follow up and deaths of suspected pneumonia cases and non-diseased subjects during the one-year follow-up.

|  | Suspected pneumonia cases | Non-diseased subjects | *p-value* |
| --- | --- | --- | --- |
| Episodes/subjects | 562 | 1,123 |  |
| Living situation at inclusion*, in % |  |  | <0.001 |
| Single household | 28.5 | 28.6 |  |
| Two or more person/household | 65.7 | 70.8 |  |
| Elderly home | 1.2 | 0.4 |  |
| Rehabilitation-/nursing home | 4.6 | 0.2 |  |
| Missing | - | - |  |
| Living situation of survivors at 12-month follow-up, in % |  |  | ns |
| Single household | 21.9 | 25.5 |  |
| Two or more person/household | 57.3 | 62.8 |  |
| Elderly home | 1.2 | 0.7 |  |
| Rehabilitation-/ nursing home | 0.4 | 0.2 |  |
| Missing/loss-to-follow-up | 19.2 | 10.8 |  |
| Died during 1-year follow, in % | 8.4 | 1.2 | <0.001 |
| Death causes, in % |  |  | 0.054 |
| Infectious diseases | 2.1 | 7.1 |  |
| Chronic lung diseases | 27.7 | 0.0 |  |
| Cancer | 17.0 | 42.9 |  |
| Cardiovascular events and stroke | 36.2 | 21.4 |  |
| Other causes | 17.0 | 28.6 |  |
| Loss-to-follow-up for other reasons than death, in % |  |  | ns |
| Bad health | 34.0 | 24.3 |  |
| Other reasons | 27.7 | 28.8 |  |
| Reason unknown | 38.3 | 45.9 |  |
| Not being able to get in contact again | - | 0.9 |  |

* At inclusion in cohort (= day of home visit).

Used abbreviations: ns = not significant;


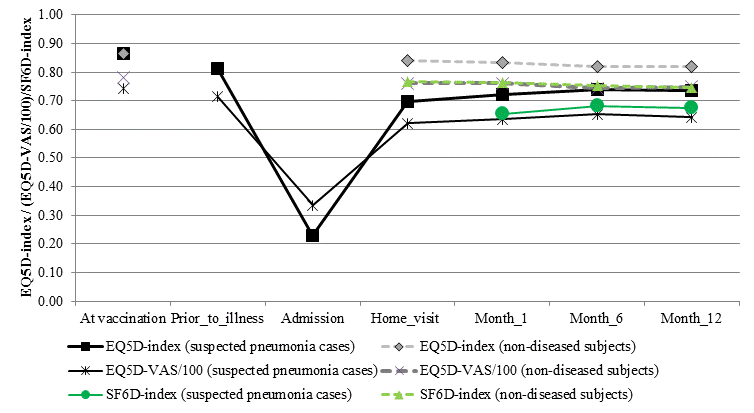
Figure A.3 – Mean EQ-5D-3L-index, EQ-VAS and SF6D-index at different contact moments for the suspected CAP cases and the non-diseased subjects, respectively.

Table A.3 – Spearman’s rho for EQ-VAS, EQ5D-index and SF6D-index at the different contact moments for suspected pneumonia cases.

|  | EQ-VAS_B_ | EQ5D-index_B_ | EQ-VAS_P_ | EQ5D-index_P_ | EQ-VAS_A_ | EQ5D-index_A_ | EQ-VAS_0_ | EQ5D-index_0_ | EQ-VAS_1_ | EQ5D-index_1_ | SF6D-index_1_ | EQ-VAS_6_ | EQ5D-index_6_ | SF6D-index_6_ | EQ-VAS_612_ | EQ5D-index_12_ | SF6D-index_12_ |
| --- | --- | --- | --- | --- | --- | --- | --- | --- | --- | --- | --- | --- | --- | --- | --- | --- | --- |
| EQ-VAS_B_ | **1** |  |  |  |  |  |  |  |  |  |  |  |  |  |  |  |  |
| EQ5D-index_B_ | **.549**** | **1** |  |  |  |  |  |  |  |  |  |  |  |  |  |  |  |
| EQ-VAS_P_ | .436** | .351** | **1** |  |  |  |  |  |  |  |  |  |  |  |  |  |  |
| EQ5D-index_P_ | .408** | .502** | **.611**** | **1** |  |  |  |  |  |  |  |  |  |  |  |  |  |
| EQ-VAS_A_ | .053 | .043 | .006 | 0.11 | **1** |  |  |  |  |  |  |  |  |  |  |  |  |
| EQ5D-index_A_ | .062 | .147* | .027 | .192** | **.453**** | **1** |  |  |  |  |  |  |  |  |  |  |  |
| EQ-VAS_0_ | .358** | .274** | .423** | .302** | .413** | .221** | 1 |  |  |  |  |  |  |  |  |  |  |
| EQ5D-index_0_ | .293** | .412** | .247** | .470** | .207** | .366** | .540** | 1 |  |  |  |  |  |  |  |  |  |
| EQ-VAS_1_ | .419** | .357** | .402** | .323** | .182** | .150** | .516** | .468** | 1 |  |  |  |  |  |  |  |  |
| EQ5D-index_1_ | .366** | .490** | .350** | .483** | .131** | .222** | .402** | .648** | .643** | 1 |  |  |  |  |  |  |  |
| SF6D-index_1_ | .322** | .421** | .340** | .400** | .142** | .193** | .416** | .566** | .623** | .720** | 1 |  |  |  |  |  |  |
| EQ-VAS_6_ | .450** | .413** | .433** | .445** | .132** | .146* | .447** | .438** | .666** | .536** | .552** | 1 |  |  |  |  |  |
| EQ5D-index_6_ | .364** | .490** | .348** | .478** | .155** | .199** | .367** | .545** | .549** | .681** | .602** | .687** | 1 |  |  |  |  |
| SF6D-index_6_ | .376** | .476** | .396** | .520** | .111* | .186** | .356** | .545** | .589** | .638** | .664** | .727** | .776** | 1 |  |  |  |
| EQ-VAS_612_ | .446** | .392** | .420** | .430** | .128** | .134** | .420** | .432** | .638** | .526** | .501** | .741** | .596** | .642** | 1 |  |  |
| EQ5D-index_12_ | .394** | .521** | .389** | .520** | .009 | .128** | .313** | .521** | .489** | .653** | .558** | .585** | .713** | .724** | .652** | 1 |  |
| SF6D-index_12_ | .385** | .478** | .378** | .483** | .065 | .141 | .362** | .480** | .536** | .599** | .586** | .628** | .677** | .761** | .679** | .806** | 1 |

Note the subscripts B, P, A, 0, 1, 6 and 12 stays for baseline (i.e. collected at vaccination); prior to illness onset (i.e. retrospectively collected at day 0); at admission (i.e. retrospectively collected at day 0); day 0 (i.e. home visit = inclusion); 1-month after home visit; 6-months after home visit and 12-months after home visit, respectively. All correlations were significant (p>0.001).

Table A.4 – Spearman’s rho for EQ-VAS, EQ5D-index and SF6D-index at the different contact moments for non-diseased subjects.

|  | EQ-VAS_B_ | EQ5D-index_B_ | EQ-VAS_0_ | EQ5D-index_0_ | SF6D-index_0_ | EQ-VAS_1_ | EQ5D-index_1_ | SF6D-index_1_ | EQ-VAS_6_ | EQ5D-index_6_ | SF6D-index_6_ | EQ-VAS_612_ | EQ5D-index_12_ | SF6D-index_12_ |
| --- | --- | --- | --- | --- | --- | --- | --- | --- | --- | --- | --- | --- | --- | --- |
| EQ-VAS_B_ | **1** |  |  |  |  |  |  |  |  |  |  |  |  |  |
| EQ5D-index_B_ | .594** | **1** |  |  |  |  |  |  |  |  |  |  |  |  |
| EQ-VAS_0_ | .615** | .477** | **1** |  |  |  |  |  |  |  |  |  |  |  |
| EQ5D-index_0_ | .465** | .615** | .560** | **1** |  |  |  |  |  |  |  |  |  |  |
| SF6D-index_0_ | .522** | .539** | .586** | .673** | **1** |  |  |  |  |  |  |  |  |  |
| EQ-VAS_1_ | .674** | .538** | .750** | .567** | .624** | 1 |  |  |  |  |  |  |  |  |
| EQ5D-index_1_ | .507** | .664** | .533** | .723** | .656** | .651** | 1 |  |  |  |  |  |  |  |
| SF6D-index_1_ | .529** | .569** | .561** | .618** | .741** | .686** | .732** | 1 |  |  |  |  |  |  |
| EQ-VAS_6_ | .692** | .522** | .673** | .541** | .562** | .764** | .570** | .615** | 1 |  |  |  |  |  |
| EQ5D-index_6_ | .506** | .641** | .516** | .666** | .619** | .605** | .757** | .673** | .677** | 1 |  |  |  |  |
| SF6D-index_6_ | .545** | .580** | .548** | .603** | .691** | .637** | .655** | .744** | .718** | .774** | 1 |  |  |  |
| EQ-VAS_612_ | .633** | .512** | .653** | .514** | .547** | .747** | .569** | .612** | .782** | .629** | .662** | 1 |  |  |
| EQ5D-index_12_ | .461** | .608** | .477** | .624** | .577** | .547** | .708** | .620** | .581** | .753** | .659** | .665** | 1 |  |
| SF6D-index_12_ | .517** | .558** | .515** | .552** | .653** | .591** | .614** | .702** | .624** | .666** | .758** | .718** | .755** | 1 |

Note the subscripts B, 0, 1, 6 and 12 stays for baseline (i.e. collected at vaccination); day 0 (i.e. home visit = inclusion); 1-month after home visit; 6-months after home visit and 12-months after home visit, respectively. All correlations were significant (p>0.001).

Table A.5 – Baseline characteristics of radiologically confirmed and non-confirmed CAP cases and their non-diseased subjects.

|  | Radiologically confirmed CAP cohort | Non-diseased subjects of radiologically confirmed CAP cases | | *p-value* | | Radiologically non-confirmed CAP cohort | Non-diseased subjects of radiologically non-confirmed CAP cases | *p-value* | Radiologically confirmed CAP cases versus  radiologically non-confirmed CAP cacses  *p-value* |
| --- | --- | --- | --- | --- | --- | --- | --- | --- | --- |
| Episodes/subjects | 341 | | 682 | |  | 221 | 441 |  |  |
| *Matching criteria* |  | |  | |  |  |  |  |  |
| Male, in % | 71.8 | | 72.0 | | n.s. | 69.7 | 69.8 | ns | ns |
| Age at inclusion^a^, median (IQR) | 76 (72-82) | | 76 (72-82) | | ns | 76 (72-81) | 76 (72-81) | ns | ns |
| EQ5D-index (at vaccination), median (IQR) | 0.87 (0.80-1.00) | | 0.89 (0.80-1.00) | | ns | 0.86 (0.78-1.00) | 0.86 (0.78-1.00) | ns | ns |
| *Other criteria* |  | |  | |  |  |  |  |  |
| Number of self-reported comorbidities at  inclusion^a^, median (IQR) | 2 (1-3) | | 2 (1-3) | | <0.001 | 3 (1-4) | 2 (1-3) | <0.001 | ns |
| Education level, in % |  | |  | | <0.001 |  |  | <0.001 | ns |
| Low | 51.3 | | 36.4 | |  | 57.0 | 36.5 |  |  |
| Medium | 28.7 | | 35.6 | |  | 27.6 | 37.9 |  |  |
| High | 19.6 | | 28.0 | |  | 14.0 | 25.6 |  |  |
| Missing | 0.3 | | - | |  | 1.4 | - |  |  |
| Region, in % |  | |  | | <0.001 |  |  | <0.001 | ns |
| North | 2.9 | | 3.7 | |  | 4.1 | 4.5 |  |  |
| East | 27.6 | | 19.9 | |  | 27.1 | 16.1 |  |  |
| West | 34.3 | | 23.2 | |  | 29.0 | 25.6 |  |  |
| South | 35.2 | | 53.2 | |  | 39.8 | 53.7 |  |  |
| Living situation at vaccination, in % |  | |  | | ns |  |  | Ns | ns |
| Single household | 29.0 | | 26.0 | |  | 25.3 | 28.1 |  |  |
| Two or more person/household | 70.1 | | 73.5 | |  | 73.8 | 71.7 |  |  |
| Elderly home | 0.6 | | 0.4 | |  | 0.9 | 0.2 |  |  |
| Missing | 0.3 | | 0.1 | |  | - | - |  |  |
| Vaccinated, in % | 44.3 | | 49.7 | | ns | 54.8 | 54.4 | Ns | 0.015 |

^a^ At inclusion in cohort (= day of home visit). ^b^ According to definition; radiologically confirmed CAP cases had to have a positive X-rays and at least 2 clinical criteria. Used abbreviations: SD: Standard deviation; ns = not significant;

Table A.6 – Living situation, loss-to-follow up and mortality of radiologically confirmed and non-confirmed CAP cases and their non-diseased subjects during the one-year follow-up.

|  | Radiologically confirmed  CAP cases | Non-diseased subject of radiologically confirmed  CAP cases | *p-value* | Radiologically non-confirmed CAP cases | Non-diseased subject of radiologically non-confirmed CAP cases | *p-value* | Radiologically confirmed CAP cases vs  radiologically non-confirmed CAP cases  *p-value* |
| --- | --- | --- | --- | --- | --- | --- | --- |
| Episodes/subjects | 341 | 682 |  | 221 | 441 |  |  |
| Living situation at inclusion*, in % |  |  | <0.001 |  |  | <0.001 | ns |
| Single household | 28.4 | 28.2 |  | 28.5 | 29.3 |  |  |
| Two or more person/household | 64.8 | 70.5 |  | 67.0 | 70.3 |  |  |
| Elderly home | 1.2 | 0.4 |  | 1.4 | 0.5 |  |  |
| Rehabilitation-/ nursing home | 5.6 | 0.3 |  | 3.2 | - |  |  |
| Missing | - | 0.6 |  | - | - |  |  |
| Living situation of survivors at 12-month follow-up, in % | 89.7 | 98.4 | ns | 94.6 | 99.3 | ns | ns |
| Single household | 21.9 | 25.5 |  | 19.1 | 26.7 |  |  |
| Two or more person/household | 57.3 | 62.8 |  | 57.9 | 60.5 |  |  |
| Elderly home | 1.2 | 0.7 |  | 1.0 | 0.7 |  |  |
| Rehabilitation- / nursing home | 0.4 | 0.2 |  | 0.5 | 0.2 |  |  |
| Missing/loss-to-follow-up | 19.2 | 10.8 |  | 21.5 | 11.9 |  |  |
| Died during 1-year follow, in % | 10.3 | 1.6 | <0.001 | 5.4 | 0.7 | <0.001 | 0.043 |
| Death causes, in % |  |  | 0.030 |  |  | ns | ns |
| Infectious diseases | 2.9 | 9.1 |  | 0 | 0.0 |  |  |
| Chronic lung diseases | 34.4 | 0.0 |  | 8.3 | 0.0 |  |  |
| Cancer | 14.3 | 54.5 |  | 25.0 | 0.0 |  |  |
| Cardiovascular events and stroke | 31.4 | 18.2 |  | 50.0 | 33.3 |  |  |
| Other causes | 17.1 | 18.2 |  | 16.7 | 66.7 |  |  |
| Loss-to-follow-up for other reasons than death, in % | 15.0 | 8.9 | ns | 19.5 | 11.3 | ns | ns |
| Bad health | 34.0 | 24.3 |  | 41.9 | 30.0 |  |  |
| Other reasons | 27.7 | 28.8 |  | 20.9 | 22.0 |  |  |
| Reason unknown | 38.3 | 45.9 |  | 37.2 | 46.0 |  |  |
| Not being able to get in contact again | - | 0.9 |  | - | 2.0 |  |  |

* At inclusion in cohort (= day of home visit). Used abbreviations: ns = not significant;

Table A.7 – EQ5D-index, EQ-VAS and SF6D-index for the radiologically confirmed and non-confirmed CAP cases and their non-diseased subjects

|  | Radiologically confirmed CAP  cases | | | | Non-diseased subject of radiologically confirmed CAP cases | | *p-value* | | Radiologically non-confirmed CAP cases | | Non-diseased subject of radiologically non-confirmed CAP cases | | *p-value* | Radiologically confirmed CAP cases vs radiologically  non-confirmed CAP cases  *p-value* |
| --- | --- | --- | --- | --- | --- | --- | --- | --- | --- | --- | --- | --- | --- | --- |
|  | Mean (SD) / *Median (IQR)* | | missing /died | | Mean (SD) / *Median (IQR)* | missing /died | |  | Mean (SD) / *Median (IQR)* | missing /died | Mean (SD) / *Median (IQR)* | missing /died |  |  |
| **EQ5D-index** |  | |  | |  |  | |  |  |  |  |  |  |  |
| At vaccination^a,b^ | 0.87 (0.16) / *0.87 (0.80-1.00)* | | 0/0 | | 0.87 (0.16) / *0.89 (0.80-1.00)* | 0/0 | | ns | 0.86 (0.16) /  *0.86 (0.78-1.00)* | 0/0 | 0.86 (0.16) */*  *0.86 (0.78-1.00)* | 0/0 | ns | ns |
| Prior to illness  onset | 0.82 (0.23) /  *0.86 (0.78-1.00)* | | 0/0 | | - | - | | - | 0.80 (0.22) /  *0.84 (0.77-1.00)* | 0/0 | - | - | - | ns |
| At admission | 0.23 (0.32) /  *0.24(-0.00-0.40)* | | 0/0 | | - | - | | - | 0.23 (0.33) /  *0.23 (-0.00-0.44)* | 0/0 | - | - | - | ns |
| During home visit | 0.70 (0.26) /  *0.78 (0.52-0.89)* | | 0/0 | | 0.85 (0.18) / *0.84 (0.78-1.00)* | 0/0 | | <0.001 | 0.70 (0.26) /  *0.78 (0.56-0.86)* | 0/0 | 0.83 (0.19) /  *0.84 (0.78-1.00)* | 0/0 | <0.001 | ns |
| Month 1 | 0.72 (0.25) /  *0.78 (0.65-0.89)* | | 44/2 | | 0.84 (0.16) /  *0.84 (0.78-1.00)* | 22/1 | | <0.001 | 0.72 (0.23) /  *0.78 (0.65-0.86)* | 17/1 | 0.82 (0.17) /  *0.81 (0.78-1.00)* | 22/1 | <0.001 | ns |
| Month 6 | 0.75 (0.23) /  *0.78 (0.69-0.89)* | | 46/25 | | 0.83 (0.18) /  *0.84 (0.78-1.00)* | 45/3 | | <0.001 | 0.72 (0.23) /  *0.78 (0.65-0.86)* | 35/8 | 0.81 (0.19) /  *0.81 (0.72-1.00)* | 31/2 | <0.001 | ns |
| Month 12 | 0.76 (0.23) /  *0.81 (0.69-0.89)* | | 57/35 | | 0.83 (0.18) /  *0.84 (0.78-1.00)* | 69/11 | | <0.001 | 0.70 (0.25) /  *0.78 (0.65-0.86)* | 49/12 | 0.80 (0.19) /  *0.81 (0.73-1.00)* | 55/3 | <0.001 | 0.008 |
| **EQ5D-VAS** |  | |  | |  |  | |  |  |  |  |  |  |  |
| Prior to illness  onset | 72 (15.4) /  *70 (65-80)* | | 1/0 | | - | - | | - | 70 (15.2) /  *70 (60-80)* | 0/0 | - | - | - | ns |
| At admission | 32 (16.0) /  *30 (20-40)* | | 1/0 | | - | - | | - | 35 (17.5) /  *35 (20-50)* | 0/0 | - | - | - | ns |
| During home visit | 62 (15.3) /  *64 (50-70)* | | 0/0 | | 76 (12.8) /  *80 (70-85)* | 3/0 | | <0.001 | 63 (15.0) /  *65 (50-70)* | 0/0 | 76 (13.8) /  *80 (70-85)* | 0/0 | <0.001 | ns |
| Month 1 | 63 (16.4) /  *65 (50-75)* | | 45/2 | | 76 (13.9) /  *80 (70-85)* | 19/1 | | <0.001 | 64 (16.2) /  *66 (50-75)* | 18/1 | 76 (13.9) /  *78 (70-85)* | 18/1 | <0.001 | ns |
| Month 6 | 66 (16.0) /  *70 (59-78)* | | 46/25 | | 74 (15.0) /  *77 (65-85)* | 42/3 | | <0.001 | 64 (17.0) /  *69 (50-79)* | 36/8 | 74 (15.7) /  *75 (67-85)* | 30/2 | <0.001 | ns |
| Month 12 | 66 (16.9) /  *70 (55-88)* | | 57/35 | | 75 (14.3) /  *76 (67-85)* | 68/11 | | <0.001 | 62 (18.2) /  *65 (50-75)* | 46/12 | 75 (15.2) /  *75 (70-85)* | 54/3 | <0.001 | ns |
| **SF6D-index** | | | | | | | | | | | | | | |
| During home visit | | - | | - | 0.78 (0.13) /  *0.80 (0.68-0.88)* | 8/0 | | <0.001 | - | - | 0.76 (0.13) /  *0.76 (0.65-0.86)* | 1/0 | - | ns |
| Month 1 | | 0.65 (0.13) /  *0.63 (0.58-0.73)* | | 55/2 | 0.77 (0.13) /  *0.80 (0.66-0.88)* | 38/1 | | <0.001 | 0.66 (0.14) /  *0.63(0.58-0.74)* | 27/1 | 0.75 (0.13) /  *0.75 (0.64-0.88)* | 31/1 | <0.001 | ns |
| Month 6 | | 0.69 (0.14) /  *0.67 (0.60-0.80)* | | 54/25 | 0.76 (0.14) /  *0.78 (0.64-0.88)* | 67/3 | | 0.001 | 0.67 (0.14) /  *0.65 (0.58-0.77)* | 41/8 | 0.74 (0.14) /  *0.73 (0.63-0.88)* | 48/2 | <0.001 | ns |
| Month 12 | | 0.68 (0.14) /  *0.68 (0.59-0.79)* | | 69/35 | 0.76 (0.13) /  *0.75 (0.64-0.88)* | 85/11 | | <0.001 | 0.66 (0.14) /  *0.65 (0.56-0.75)* | 55/12 | 0.74 (0.14) /  *0.73 (0.62-0.84)* | 64/3 | <0.001 | ns |

^a^ matching criterion

Used abbreviations: ns = not significant;

Figure A.4 – Profile of the population using EQ5D-instrument: Percentage reporting any problems per domain at different contact moments for A) the radiologically confirmed CAP cases and their non-diseased subjects, and B) the radiologically non-confirmed CAP cases and their non-diseased subjects, respectively.

| A.) Radiologically confirmed CAP cases and their non-diseased subjects | B.) Radiologically non-confirmed CAP cases and their non-diseased subjects |
| --- | --- |
| 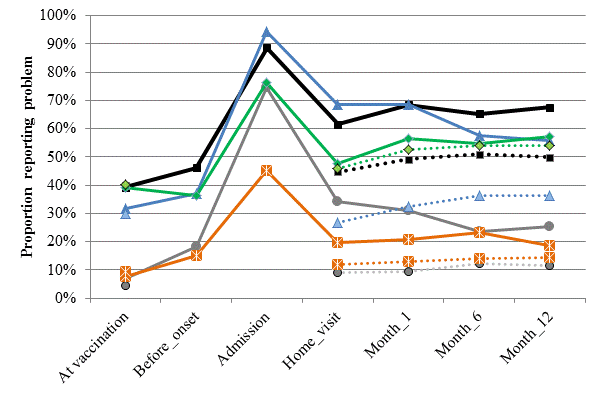 | 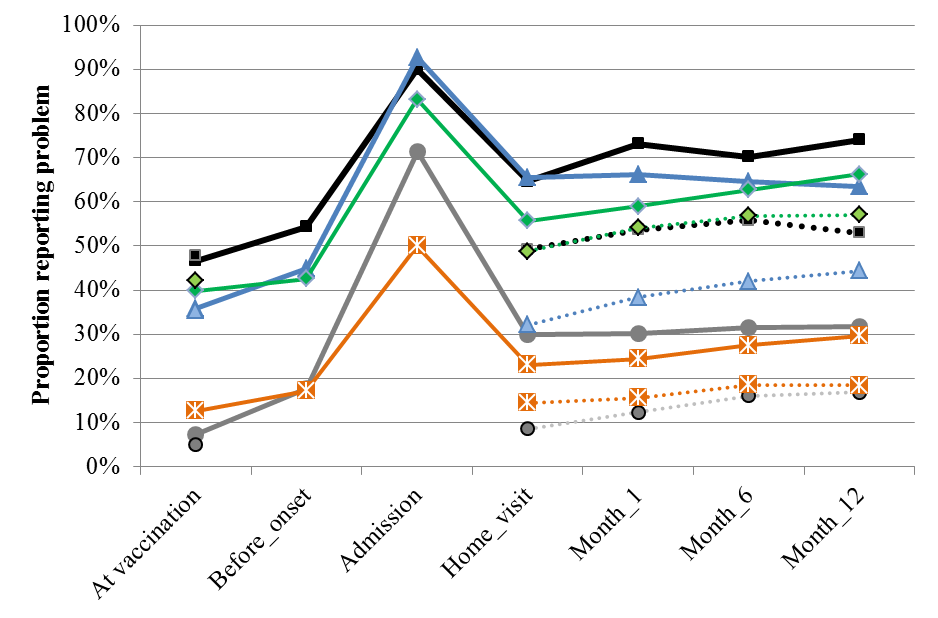 |
| 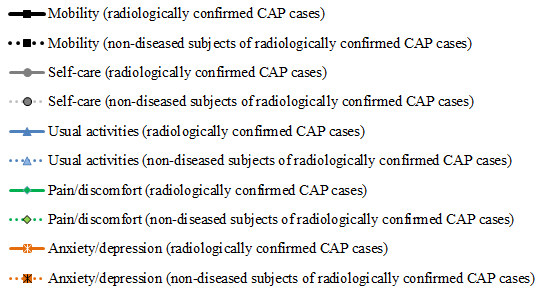 | 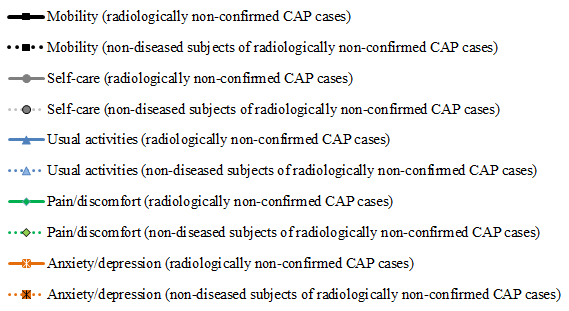 |

Note: No significant difference at baseline in any domain. Significant differences (p<0.05) for all domains on all contact moments during the follow-up period.

Figure A.5 – SF-36 mean scale scores at different contact moments for A) the radiologically confirmed CAP cases and their non-diseased subjects, and B) the radiologically non-confirmed CAP cases and their non-diseased subjects, respectively.

| A.) Radiologically confirmed CAP cases and their non-diseased subjects | B.) Radiologically non-confirmed CAP cases and their non-diseased subjects |
| --- | --- |
| 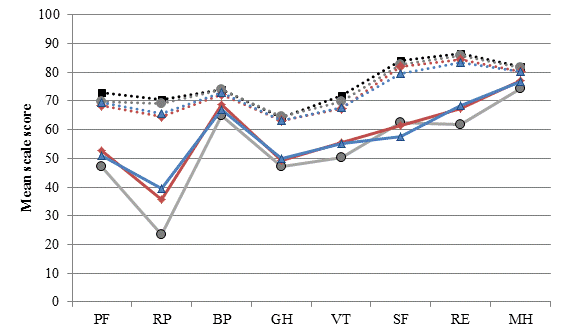 | 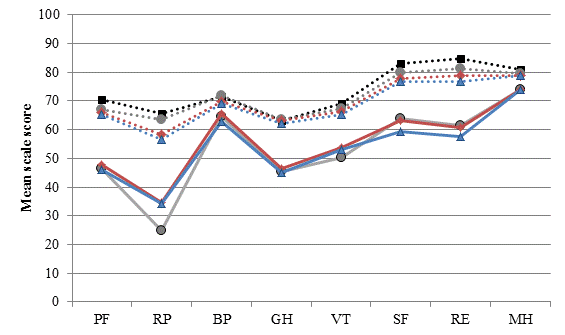 |
| 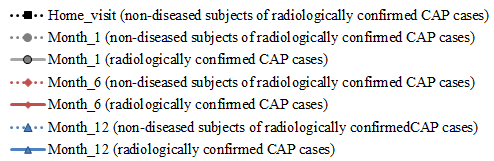 | 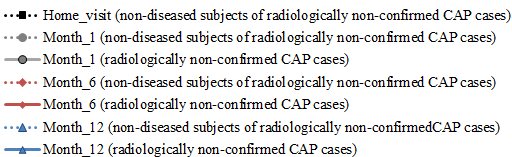 |

Abbreviations: PF=Physical Function; RP=Role-Physical; BP=Bodily Pain; GH=General Health; VT=Vitality; SF=Social Functioning; RE=Role-Emotional; MH=Mental Health. Note: Significant differences (p<0.05) for all domains on all contact moments during the follow-up period. The SF-36 survey was administered in radiologically confirmed and non-confirmed CAP cases at month 1, at month 6 and at month 12, and in non-diseased subjects during the home visit, at month 1, at month 6 and at month 12.

Figure A.6 – Mean EQ-5D-3L-index, EQ-VAS and SF6D-index at different contact moments for the radiologically confirmed CAP cases and their non-diseased subjects (A), and for the radiologically non-confirmed CAP cases and their non-diseased subjects (B)

| A.) | B.) |
| --- | --- |
| Radiologically confirmed CAP cases and their non-diseased subjects | Radiologically non-confirmed CAP cases and their non-diseased subjects |
| 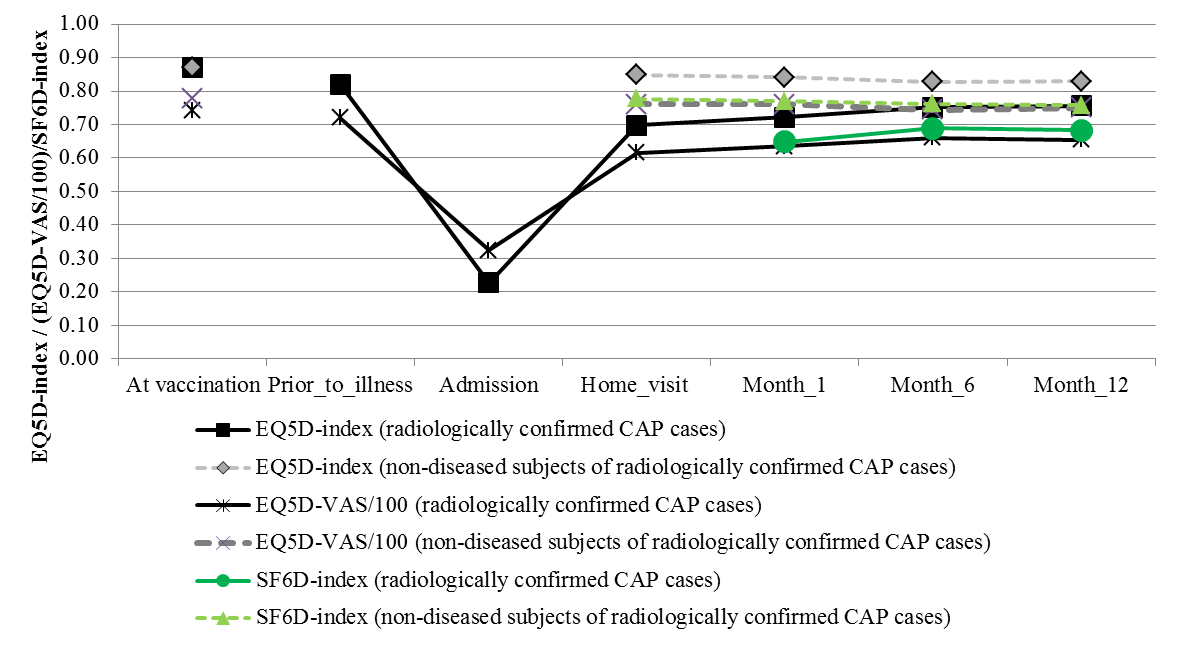 | 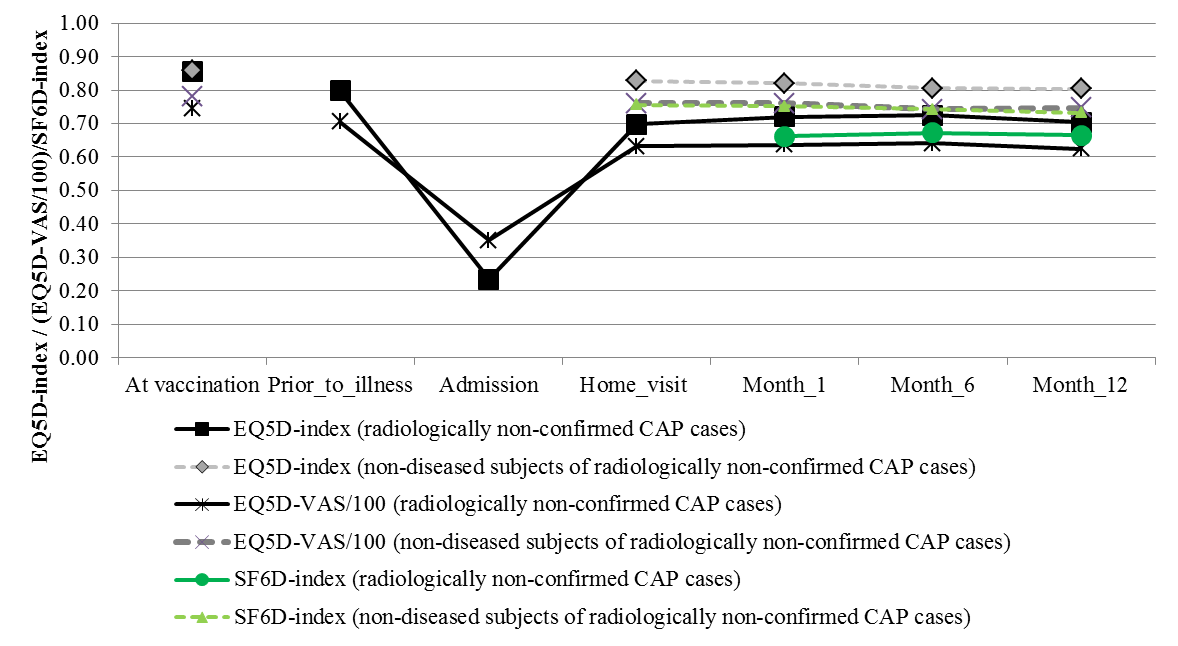 |

Note: The SF6D was derived from the SF-36 survey, which was only administered in radiologically confirmed and non-confirmed CAP cases at month 1, at month 6 and at month 12, and in non-diseased subjects during the home visit, at month 1, at month 6 and at month 12

Table A.8 –Utility differences attributable to radiologically confirmed CAP and radiologically non-confirmed CAP, respectively.

|  | | QALY of  radiologically confirmed CAP cases  (SE) | QALY of  non-diseased subjects of radiologically confirmed CAP cases  (SE) | Utility difference attributable to radiologically confirmed CAP | QALY of  radiologically non-confirmed CAP cases  (SE) | QALY of non-diseased subjects of radiologically non- confirmed CAP cases  (SE) | Utility differences attributable to radiologically non-confirmed CAP |
| --- | --- | --- | --- | --- | --- | --- | --- |
| All cases | | | | | | | |
|  | One-year post-discharge | 0.67 (0.01) | 0.82 (0.01) | -0.14 | 0.68 (0.01) | 0.80 (0.01) | -0.12 |
|  | Pneumonia episode & one-year post-discharge | 0.66 (0.01) | 0.82 (0.01) | -0.16 | 0.67 (0.01) | 0.80 (0.01) | -0.13 |
| Only survivors | | | | | | | |
|  | One-year post-discharge | 0.72 (0.01) | 0.83 (0.01) | -0.10 | 0.70 (0.01) | 0.81 (0.01) | -0.10 |
|  | Pneumonia episode & one-year post-discharge | 0.71 (0.01) | 0.83 (0.01) | -0.11 | 0.69 (0.01) | 0.81 (0.01) | -0.11 |
